# Compartment syndrome: A rare complication of tensor fascia lata flap reconstruction following ilio-inguinal block dissection

**DOI:** 10.4103/0970-0358.44936

**Published:** 2008

**Authors:** S. Gowthaman, N. Kathiresan, B. Satheesan

**Affiliations:** Division of Genito-Urinary Oncology, Department of Surgical Oncology, Cancer Institute (W.I.A.), Adyar, Chennai-600 036, India

Sir,

We present here a rare instance of compartment syndrome following ilio-inguinal block dissection (IIBD) with a tensor fascia lata flap (TFL flap) reconstruction done for a patient with penile cancer. In our center, we use a TFL flap for cover for large cutaneous defects following IIBD.

A 49 year-old male patient with squamous cell carcinoma of the penis underwent partial penectomy on 10^th^ Jan.2007. He presented with a 4 × 3 cm inguinal nodal mass after 11 months. He underwent left IIBD with a TFL flap, right superficial inguinal dissection, and frozen section study on 9th Jan 2008.The donor site of the TFL flap was primarily closed as the width of the flap was 6 cm. Closure of the donor site was done by approximating the fascial margin in order to avoid wound breakdown. On the first postoperative day, he developed compartment syndrome in the left lower limb with neurovascular compromise. The entire limb was tense with absence of distal pulses and sensory-motor deficit. He did not have significant pain due to the epidural analgesia. Immediately, all donor site sutures were removed and anterolateral and posterior fasciotomies were done in the leg. Vascularity improved although there was lack of sensory-motor function in the lower limb. Debridement of the vastus lateralis and intermedius was done for superficial muscle necrosis after three days. He was mobilized from the 15^th^ postoperative day and nerve stimulation exercises were started. Motor functions and sensory deficits improved gradually and the patient is able to walk well without support now.

IIBD has traditionally been associated with a high incidence of complications.[1] The inguinal defects secondary to inguinal block dissections can be covered with a simple split skin graft, rectus abdominis flap, anterolateral thigh rotation flap, or TFL flap. A TFL flap is safe and reliable with minimal postoperative morbidity.[1] The donor site defect can be either grafted with SSG or primarily closed. If the width of the flap is < 8 cm, primary closure can be done without much difficulty. Although some close the fascia if the defect is narrower; it may be preferable to avoid this approach. The following factors are believed to have caused or contributed to the problem: i) approximation of deep fascia to close the defect might have led to the syndrome, ii) good postoperative analgesia masked the symptoms, and iii) the syndrome is literally unheard of as a complication of the TFL flap and the authors have never come across such situation in their clinical practice. The latter two might be the reasons for delayed detection. The complication may be due to the technical flaw which we would like to highlight for the benefit of young reconstructive surgeons and those using a similar technique.
